# Innate Receptors Expression by Lung Nociceptors: Impact on COVID-19 and Aging

**DOI:** 10.3389/fimmu.2021.785355

**Published:** 2021-12-16

**Authors:** Carlos H. Hiroki, Nicole Sarden, Mortaza F. Hassanabad, Bryan G. Yipp

**Affiliations:** ^1^ Calvin, Phoebe and Joan Snyder Institute for Chronic Diseases, Cumming School of Medicine, University of Calgary, Calgary, AB, Canada; ^2^ Department of Critical Care, Cumming School of Medicine, University of Calgary, Calgary, AB, Canada

**Keywords:** sensory neurons, nociceptors, innate receptors, COVID-19, aging, inflammaging, neuroimmune crosstalk

## Abstract

The lungs are constantly exposed to non-sterile air which carries harmful threats, such as particles and pathogens. Nonetheless, this organ is equipped with fast and efficient mechanisms to eliminate these threats from the airways as well as prevent pathogen invasion. The respiratory tract is densely innervated by sensory neurons, also known as nociceptors, which are responsible for the detection of external stimuli and initiation of physiological and immunological responses. Furthermore, expression of functional innate receptors by nociceptors have been reported; however, the influence of these receptors to the lung function and local immune response is poorly described. The COVID-19 pandemic has shown the importance of coordinated and competent pulmonary immunity for the prevention of pathogen spread as well as prevention of excessive tissue injury. New findings suggest that lung nociceptors can be a target of SARS-CoV-2 infection; what remains unclear is whether innate receptor trigger sensory neuron activation during SARS-CoV-2 infection and what is the relevance for the outcomes. Moreover, elderly individuals often present with respiratory, neurological and immunological dysfunction. Whether aging in the context of sensory nerve function and innate receptors contributes to the disorders of these systems is currently unknown. Here we discuss the expression of innate receptors by nociceptors, particularly in the lungs, and the possible impact of their activation on pulmonary immunity. We then demonstrate recent evidence that suggests lung sensory neurons as reservoirs for SARS-CoV-2 and possible viral recognition *via* innate receptors. Lastly, we explore the mechanisms by which lung nociceptors might contribute to disturbance in respiratory and immunological responses during the aging process.

## Introduction

### The Lungs Are Constantly Exposed to External and Hazardous Threats

The lungs are responsible for the process of breathing whereby gas exchange occurs between the external air and the bloodstream. Upon contraction of the diaphragm and intercostal muscles, the lungs expand, resulting in the creation of a negative pressure when compared to the external pressure (1). As a result, the air travels through the nose, oropharynx, larynx, trachea, bronchi, and spreads into the several bronchioles inside of the lungs, allowing an extensive area of contact with the alveolar epithelial cell layer (over 70m^2^) (2). For proper gas exchange, the distance between the alveolar and the vascular compartments must be thin (around 2 μm) as well as soft for efficient gas diffusion through the tissue (3). Furthermore, an average adult breathes thousands of liters of air per day (4); however, the external air is not sterile, carrying particles and pathogens. Therefore, due to the constant exposure of non-sterile air with an extensive and delicate tissue, any insult could reach the lungs and cross the epithelial and interstitial barriers, reaching the bloodstream and causing systemic inflammation. Nevertheless, the combination of lung sensory neuron-triggered reflexes, which expel these threats from the airway, along with a unique immunological niche equipped to rapidly eliminate microbes, guarantees at least some level of protection.

Pulmonary host defense relies on both immune and nervous systems. An efficient immunological milieu composed of patrolling leukocytes, such as alveolar macrophages and neutrophils, clears pathogens in the alveoli and prevents their dissemination into the bloodstream (5, 6). In addition, sensory neurons detect external threats and trigger reflexes, such as cough or sneezing, leading to their expulsion (7). These neurons are located throughout the respiratory tract (upper and lower airways) working as “mucosal guards”, alerting the central nervous system, and initiating local responses upon detection of insults (8).

## Lung Sensory Innervation

**Figure 1:**
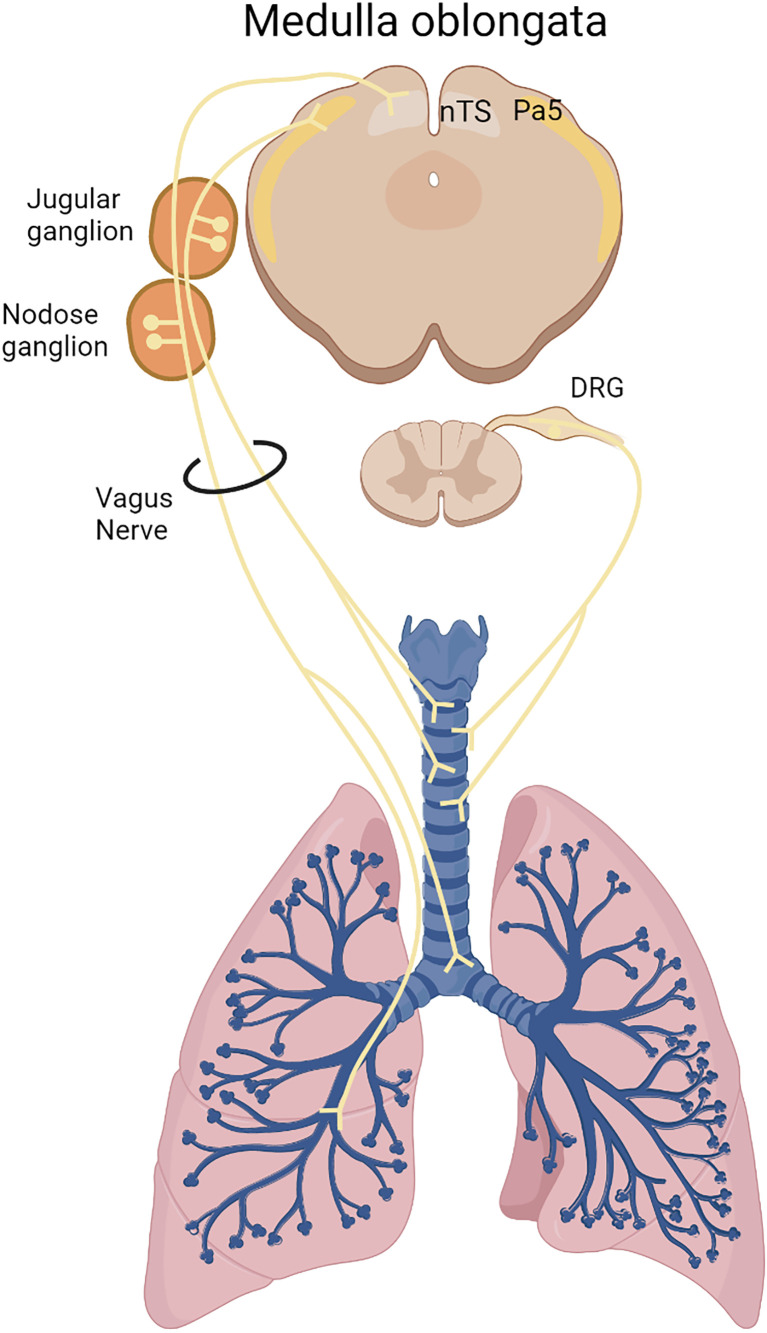
Lung sensory innervation. Sensory neurons innervate the lungs originating in the medulla oblongata *via* the vagus nerve from the nodose/jugular ganglia or from the dorsal root ganglion (DRG) in the spinal cord. Nodose neurons innervates most of intrapulmonary sites and communicates with the nucleus of the solitary tract (NTS), whereas jugular neurons innervate most of extrapulmonary sites and reaches the paratrigeminal center (Pa5).

Sensory neurons are responsible for the detection of harmful external stimuli in the lungs and in the airways, providing inputs to the central nervous system through the vagus nerve and the spinal dorsal root ganglia (DRG) (9). Most of the lung sensory neurons come from the vagal innervation, which can be stratified into the jugular and nodose ganglia. Anatomically, jugular innervation terminates mostly in extrapulmonary sites and reaches the paratrigeminal nucleus whereas nodose neurons innervate most of intrapulmonary sites and communicate with the nucleus of the solitary tract (8, 10). This afferent function informs the central nervous system, which in response, coordinates a physiological response. Furthermore, sensory neurons also perform an efferent function through the release of neuropeptides, which triggers local responses (11). A representation of the lung sensory innervation is demonstrated by the **Figure 1**.

Sensory neurons can be categorized as A-fibers and C-fibers based on physiological properties, production of neuropeptides and speed of conduction. A-fibers are non-peptidergic, myelinated and conduct the electrical pulse with a speed of 5-18m/s (10). Their terminal innervation ends in airway smooth muscle cells and neuroepithelial bodies in the airway epithelium, where they are responsible for the detection of mechanostimuli, such as lung inflation as well as chemical and thermal stimuli (12). C-fibers are unmyelinated and slow-conducting neurons (impulse speed is around 1 m/s) responsible for the detection of thermal, physical and chemical stimuli, such as capsaicin and bradykinin, from extrapulmonary (larynx, trachea and main bronchi) and intrapulmonary tissue (13). Most of the C-fiber neurons express transient receptor potential (TRP) cation channels, such as TRPA1 and TRPV1. These receptors are responsible for the detection of heat, acidosis, osmosis, cold and environmental irritants (14). A summary of the cellular a physiological functions of A- and C- fibers are summarized at **Table 1**. Upon activation, a calcium influx triggers neuron depolarization, resulting in the action potential firing and releasing the feeling of pain or spiciness to the central nervous system as well as inducing reflexes, such as cough and sneezing (15). Importantly, TRP channels are not alone in their ability trigger sensory neuron activation. Innate receptors present on the cell surface as well as in the cytoplasm allows sensory neuronal activation upon recognition of pathogens or damage-associated molecular patterns (PAMPs and DAMPs, respectively).

**Table 1:** Summary of the key differences and similarities between A- and C-fibers.

	C-fibers	A-fibers
**Fiber Subtypes**	N/A	Aβ-, Aδ-fibers
**Myelination & Diameter**	Unmyelinated and 0.2-1.5 μm	Myelinated and 7-20 μm
**Conduction Speeds**	0.5 – 2 m/s	5 – 20 m/s
**Stimulus**	Chemical Stimuli (eg. Capsaicin, Acidity), Osmolarity, Temperature changes	Mechanical forces (tissue stretch, punctate stimuli), Acidity, ATP
**Anatomical Connections**	Intrapulmonary and Extrapulmonary terminations ➔ Dorsal Root Ganglion & afferent port of Vagus Nerve (Jugular Ganglion, Nodose Ganglion) ➔ CNS	
**Physiological Response**	Cough, Bronchoconstriction, Tachypnea	Cough, Bronchoconstriction & Bronchodilation, Tachypnea

N/A, Not applicable.

## Innate Receptors Expression by Nociceptors

**Figure 2:**
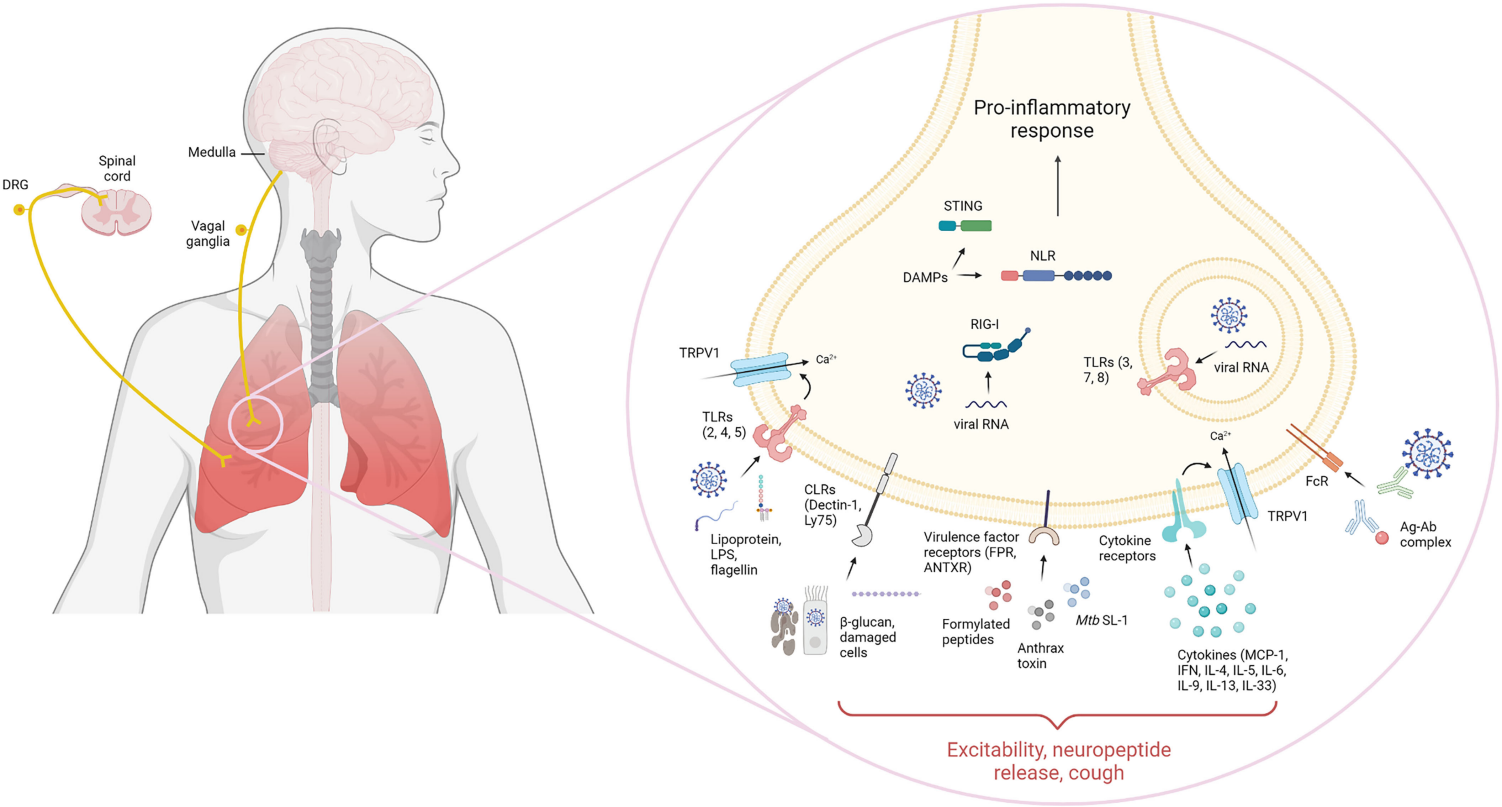
Lung sensory innervation and innate receptor expression. Lung sensory neurons express a variety of innate receptors that interact with their ligands to sense pathogen/damage-associated molecular patterns (PAMP/DAMP). Nociceptors in the dorsal root ganglion and nodose/jugular ganglia are known to express functional extracellular and intracellular Toll-like receptors (TLR). These include TLR2, which recognize bacterial lipoproteins, TLR4 which senses LPS and TLR5 which recognizes flagellin. Intracellularly, TLR3, 7 and 8 can recognize double-stranded and single-stranded RNA. Additionally, nociceptors have been shown to couple TLRs non-canonically to ion-channels such as TRPV1. Sensory neurons can also express C-type lectin receptors, including Dectin-1 and Ly75 that could sense β-glucan or apoptotic and necrotic cells, respectively. Direct recognition of microbial virulence factors is also known to occur in nociceptors. These include expression of formyl peptide receptors which bind N-formylated peptides, anthrax toxin through binding to anthrax toxin receptor and *M. tuberculosis*-derived sulfolipid-1 through an unknown neuronal receptor. Intracellularly, nociceptors can also express intracellular innate sensors, such as cytosolic DNA sensors, retinoic acid-inducible gene I and NOD-like receptors which recognize danger-associated molecular patters and nucleic acids in the cytoplasm. Receptors for immune mediators have also been found in nociceptors, including cytokine receptors recognizing MCP-1, type I and II interferon, IL-4, IL-5, IL-6, IL-9, IL-13 and IL-33 as well as antibody receptors, including FcϵR and FcγR, recognizing antigen-complexed IgE and IgG, respectively. Nociceptor sensing of PAMPs/DAMPs leads to activation and initiation of physiological and immunological responses.

Upon infection and/or injury, immune cells respond to PAMPs/DAMPs through widely conserved pathogen-recognition receptors (PRRs) such as toll-like receptors (TLR), C-type lectin receptors (CLR), Nod-like receptors (NLR), RIG-I-like receptors (RLR) and cytosolic DNA sensors (CDS). It has become evident that nociceptors can directly recognize pathogens and danger signals by their expression of innate receptors, which can result in pain, itch, hypernociception or in the context of lung nociceptors, the induction of cough. Additionally, sensory neurons can detect immune molecules such as cytokines and antibodies through expression of their respective receptors. Given the abundant distribution of sensory neurons across the lung, they are one of the first cells exposed to insults and their expression of innate receptors poise them for immediate modulation of an inflammatory response. A representation of innate receptors expression in nociceptors is demonstrated by **Figure 2**.

### Toll-Like Receptors

Toll-like receptors are the best characterized PRR. Each TLR is composed of leucine-rich repeats that mediate recognition, a transmembrane domain and a cytoplasmic domain that initiates downstream signaling (16). TLRs interact with their ligand as a homo or heterodimer which leads to activation of intracellular signaling pathways including MyD88, NF-κB and MAP kinase pathways resulting in the initiation of an inflammatory response (16). TLRs include 10 receptors in humans (TLR 1-10) and 12 in mice (TLR 1-9, 11-13). TLRs localize in the cell surface or intracellular compartments and recognize various PAMPs. For instance, TLR1 and 2 recognize bacterial lipoproteins, TLR3 recognizes double stranded RNA (dsRNA), TLR4 recognizes LPS, TLR5 recognizes flagellin, TLR7 and 8 sense single stranded RNA and TLR9 recognizes unmethylated CpG motifs (17).

Nociceptors in the DRG and nodose/jugular ganglia are known to express functional TLRs, including TLR2, TLR3, TLR4, TLR5, TLR7, TLR8 and TLR9, extensively reviewed elsewhere (18). Nociceptors have also been shown to couple TLRs non-canonically to ion-channels (19, 20). Interestingly, using transcriptomic profiling in nociceptors, TLR4 has found to be the most highly expressed PRR (18). Lipopolysaccharide (LPS) from gram-negative bacteria, the classical TLR4 ligand, is known to directly activate nociceptors through TLR4 resulting in TRPV1 sensitization, calcium influx and neuropeptide release (20). For example, LPS stimulates CGRP release from the vagal ganglia through TLR4 expression in vagal afferents (21). Furthermore, LPS is also known to induce pelvic pain in the context of urinary tract infections in a TLR4-dependent manner (22). In addition, TLR4 expression in nociceptors drove the development of neuropathic pain in female mice, resulting in mechanical hypersensitivity while absent in conditionally depleted mice (23). Moreover, LPS stimulation has been shown to depend on TRPA1 channels as LPS-induced calcium influx, membrane depolarization and neuropeptide release were absent in TRPA1 deficient mice (24). Similarly, poly(I:C), a dsRNA analogue which binds TLR3, induced inward currents and action potentials in DRG neurons resulting in itch which was absent in TLR3 deficient mice. Notably, the microRNA miRNA-let-7b, a ligand of TLR7 in mouse DRG neurons has been shown to activate nociceptors through coupling of TLR7 and TRPA1 which elicited pain (25). TLR5 has also been shown to be activated by flagellin in DRG nociceptors and addition of QX-314, a membrane impermeable analgesic, leads to TLR5-dependent blockade of neuropathic pain (26). MyD88, the common and essential intracellular signaling molecule for all TLR, has been shown to be present in the majority of DRG neurons suggesting functionality of TLRs and the ability to signal through canonical TLR signaling (27, 28). MyD88 activation can lead to neuronal excitation and the production of neuropeptides that can modulate the inflammatory response. Conditional depletion of MyD88 in nociceptors resulted in reduced IL-1β-induced pain and immune cell infiltration (28). In the lung, a study detected expression of TLR2 and TLR5 in pulmonary sensory neurons using RT-PCR which might be relevant in the context of pulmonary infections (29). Thus, an expanding body of literature highlights that sensory neurons express functionally relevant detection systems of the TLR pattern recognition pathways.

### CLRs

C-type lectin receptors (CLRs) are carbohydrate-binding innate receptors which bind carbohydrate moieties using conserved carbohydrate recognition domains (30). In immune cells, CLRs are important for recognition and binding to pathogens resulting in internalization, degradation, and antigen presentation (30). Some CLRs include the mannose receptor (MR), DC-associated C-type lectin (Dectin-1) and DC-specific ICAM3 grabbing non-integrin (DC-SIGN). In nociceptors, expression of CLRs remains incompletely understood. Fungal-derived β-glucan was shown to stimulate nociceptors dependent on Dectin-1 allowing detection of the *Candida albicans* cell wall. Notably, Dectin-1-deficient mice were unresponsive to pain induced by Candidiasis suggesting Dectin-1-dependent recognition of fungal components (31). Furthermore, transcriptomic profiling of DRG nociceptor cells revealed high expression of Ly75 in peptidergic nociceptors (18, 32). Ly75 (also known as DEC205) has been suggested to bind apoptotic and necrotic cells (33); yet, it remains unclear if functional Ly75 is indeed present on nociceptors and the functional outcomes of their expression. Ly75 could promote sensing of damage to host cells upon inflammation or infection resulting in nociceptor activation and neuropeptide release. In addition, during viral infection where virus-infected cells become apoptotic, having sensors of apoptotic and necrotic cells could modulate the inflammatory response. In the lung specifically, expression of Dectin-1 or LY75 in nociceptors could be beneficial upon infectious insults that would allow lung nociceptors to sense either direct microbial structures, such as fungal β-glucan, or apoptotic and necrotic cells to steer the immune response.

### Virulence Factor Sensing

Peripheral nerves can also directly recognize and become activated by microbes due to expression of receptors that can recognize microbial virulence factors. *Staphylococcus aureus*, a human pathogen relevant for pulmonary infections, contains virulence factors that can directly activate nociceptors. For example, DRG and trigeminal ganglia nociceptors are known to express G-protein coupled formyl peptide receptors Fpr1 and Fpr2 which bind *S. aureus*-derived N-formylated peptides (34). Furthermore, pore-forming toxins from *S. aureus* trigger sensory neurons through a TRPV1-dependent mechanism (35). Nociceptive neurons were also found to express the anthrax toxin receptor ANTXR2 which recognizes *Bacillus anthracis* derived toxins (36). Lastly, *Mycobacterium tuberculosis*-derived sulfolipid-1 was shown to activate nociceptive neurons and induce cough. It was suggested that the mechanism of cough was through direct activation of an unknown neuronal receptor recognizing sulfolipid-1 (37).

### Intracellular Innate Sensors

Intracellular innate sensors, such as cytosolic DNA sensors, RIG-I-like receptors and NOD-like receptors have been shown to be expressed in sensory neurons, however their relevance and functionality remains incompletely understood. Cytosolic DNA sensors (CDS) detect self- and pathogen-derived DNA. DNA sensing can lead to induction of type-I interferons and pro-inflammatory cytokines. In particular, stimulator of IFN genes (STING), an endoplasmic reticulum bound DNA sensor, has been recently shown to be highly expressed in peptidergic nociceptive neurons in the DRG (38, 39). Retinoic acid-inducible gene I (RIG-I)-like receptors (RLR) are nucleic acid sensors located in the cytoplasm. Nucleic acids, such as viral RNA, trigger their activation which induces the production of type I interferons and establish an anti-viral host response (40). The RLRs family include RIG-I, MDA5 and LGP2. Single-stranded RNA viruses such as coronaviruses and influenza viruses are known to be recognized by RLRs, namely RIG-I and MDA5 (41–44). Deep RNA-Seq profiling of mouse DRG neurons indicated that peptidergic nociceptor neurons exhibited high expression of *Ddx58*, the gene encoding RIG-I (18, 38). Lastly, nucleotide-binding and oligomerization (NOD)-like receptors are cytoplasmic receptors with roles in inflammasome assembly, signal transduction, transcription activation, and autophagy. scRNA-seq profiling showed expression in mouse DRG nociceptors of several NLR genes including *Nlrx1* and *Nod1* (18, 32, 38). These two non-macromolecular scaffold-forming NLRs have a role in regulation of inflammation signaling through the activation of NF-kB, MAP kinase, and interferon regulatory factors to stimulate innate immunity (45). Yet, it remains unclear what is the functional relevance of sensory neuron expression of NLR.

### Cytokine Sensing

Nociceptors have been documented to directly sense cytokines and chemokines resulting in alterations in activity and excitability. For example, CCR2, the receptor for MCP-1/CCL-2 has been found to be expressed by nociceptor neurons from DRG in models of neuropathic pain. Indeed, activation of CCR2 by MCP-1 could sensitize nociceptors *via* transactivation of TRP channels (46). Additionally, sensory neurons in the mouse DRG were shown to express allergy associated type 2 cytokine receptors such as IL-4Rα and IL-13Rα1 (47). Interestingly, sensory neuron-specific deletion of IL-4Rα reduced chronic itch, a notable symptom of allergic diseases. Alternatively, hyperalgesia was found to be associated with pro-inflammatory pathways such as the overexpression of TNF receptors in the DRG in a model of joint pain (48). Likewise, IL-6 was shown to activate nociceptors in an antigen-induced arthritis model, resulting in neuropeptide release and joint pain (49). The pro-inflammatory cytokine IL-1β can be detected by IL-1R in nociceptors in the DRG leading to activation and pain allowing nociceptors to sense tissue inflammation (50). Conversely, anti-inflammatory cytokines can also have an effect in nociceptor activation. The receptor for the anti-inflammatory cytokine IL-10 is known to be expressed in nociceptors in the DRG and can inhibit sensory neurons thereby alleviating pain (51–53). In the setting of viral infections, type I interferon receptors have been shown to be expressed in DRG neurons and type I IFNs can directly cause nociceptor sensitization (54). Relevant to the lung, transcript profiling of naive nodose ganglion revealed lung nociceptors expressed receptors for IL-33, IFN-γ, IL-4 and IL-9 and IL-5. Notably, innate lymphoid cell 2 (ILC2)-derived IL-5 directly activates sensory neurons driving allergic airway inflammation (55). Thus, during inflammation, pro-inflammatory cytokines can directly activate sensory neurons that could contribute to the amplification of the inflammatory response and effector functions of immune cells through the release of neuropeptides.

### Antibody Receptors

Antibodies can be recognized by Fc receptors present in immune cells to carry out antibody effector functions. Recent studies have elucidated that Fc-gamma receptors, recognizing IgG antibodies, are present in a subpopulation of DRG nociceptors which are activated by immune complexes and result in increased neuronal excitability (56). Interestingly, in the context of allergic airway inflammation, the high affinity Fc-epsilon receptor (FcϵR1), recognizing IgE antibodies that mediate allergic responses, is expressed in the jugular/nodose ganglion. Allergen-complexed IgE resulted in depolarization, action potential firing, calcium influx and neuropeptide release which initiated and amplified allergic airway inflammation through the release of substance P, promoting a Th2 phenotype in the airways (57). In response, substance P also induces formation of antibody producing cells as well as the release of IgE (58). The role of antibody-antigen complexes in infectious diseases, such as respiratory viral infections and the expression of Fc receptors in lung nociceptors remains elusive. However, it could be possible that antibody-virus complexes may be recognized by lung nociceptors and might trigger neuronal excitation and neuropeptide release to modulate the immune response.

Overall, despite the characterization of innate receptors function in nociceptors, few studies have characterized their relevance in the setting of lung diseases. The anatomical distribution of sensory neurons throughout the respiratory tract allows these cells to rapidly detect aspirated threats and initiate physiological as well as immunological responses.

## Nociceptor-Induced Responses in the Lungs

As discussed earlier, the upper and lower respiratory tracts are densely innervated with sensory, sympathetic, and parasympathetic fibers that work together to maintain a broad range of homeostatic functions. One such important function is the initiation of reflex arcs that remove threats such as pathogens and/or limit exposure to other noxious stimuli present in the air (59). A key example is the cough reflex arc which clears the airways of foreign objects and secretions. Interestingly, aberrant lung nociceptor function has been implicated in a variety of respiratory diseases such as chronic cough, asthma, and airway hyperreactivity (10).

Triggering tachypnea, bronchoconstriction, and increased mucus secretions, C-fibers respond to activators of TRPV1 and TRPA1 channels (capsaicin and allyl isothiocyanate, respectively), inflammatory mediators (e.g. bradykinin, prostaglandin E2), and environmental irritants (e.g. ozone, nicotine) (60). Conversely, A-fibers lack the prototypical nociceptive ion channels such as TRPV1 and TRPA1 (61). Instead, they are activated by mechanical cues such as sustained lung inflation and aspiration associated stimulus (i.e., punctate mechanical stimuli and H^+^). Upon activation, a shared physiological response of both A- and C-fibers is the cough reflex (10).

Following their stimulation, A- and C-fibers send impulses *via* the afferent pathway to the vagus nerve which relays the signal to the medulla oblongata in the brainstem (62). The central pathway (referred to as the ‘cough center’) of the medulla then produces yet another impulse which is sent through various efferent pathways (i.e. vagus, phrenic, and spinal motor nerves) to recruit the diaphragm, abdominal wall and expiratory muscles for cough production. Subsequently, the events of coughing can be divided into 3 stages: inspiratory, compression, and expiratory phase (10). During the inspiratory phase, brief inspiration ensures adequate volumes of air are present for cough production. Next, intrapulmonary pressures massively increase as the muscles of the chest wall, diaphragm, and abdominal wall contract – resulting in expiration against a closed glottis. Lastly, the expiratory phase is characterized by opening of the glottis, high expiratory velocities, and expulsion of mucus and/or foreign objects from the airways (62).

To the best of our knowledge, there are few articles discussing innate sensing of pathogens *via* nociceptors and the cough reflex. For example, *Mycobacterium tuberculosis* initiates cough *via* SL-1 (37). Furthermore, *Bordetella pertussis*, which is the causative agent of whooping cough is sensed by TLR4 found on resident and infiltrating leukocytes (63). This results in the production of strong proinflammatory mediators such as IFN-γ, nitric oxide (NO), and TNF-α. Subsequently, other studies have shown, albeit in different animal models, that application of TNF-α reduces neuronal activation thresholds and evokes A- and C-fiber depolarization (64, 65).

Another feature of sensory neuron function is the modulation of the immune response in mucosal tissues. This neuroimmune crosstalk is necessary for a proper coordination of the immune response which will efficiently combat pathogen spread, without an overwhelming inflammation, maintaining homeostasis and tissue integrity. Neuroimmunity is well described in the gut and skin, but poorly explored in the lungs. Hence, we highlight comparisons with neuroimmune interactions in the skin, where applicable.

Interestingly, neurogenic inflammation has been shown to initiate pulmonary pathological processes, such as asthma and rhinitis. Depletion or suppression of sensory neurons results in reduced allergic airway inflammation and bronchial hypersensitivity. Mechanistically, IL-5-activated sensory neurons release neuropeptides, such as VIP and NMU, which induce ILCs to produce type 2 cytokines, such as IL-5 and IL-13, contributing to the allergic response (55, 66). On the other hand, CGRP leads to regulation of ILC2-derived type 2 cytokines, demonstrating that distinct release of neuropeptides drives different outcomes in ILC2-mediated airway hypersensitivity responses (67). In the setting of allergic responses in the skin, upon cutaneous allergen-exposure sensory neurons are activated and release Substance P which stimulates dendritic cell migration to the lymph node and promotes Th2 differentiation and the initiation of an allergic response (68). Similarly, in a model of atopic dermatitis, allergen activated TRPV1+ nociceptors drove the development of allergic skin inflammation through production of Substance P which triggered mast cell degranulation (69). On the other hand, sensory neurons can also restrain inflammation and support healing by promoting a reparative phenotype in macrophages in a sunburn-like mouse model. Release of the neuropeptide TAFA4 promoted macrophage production of IL-10 which reduced skin inflammation, promoted tissue regeneration, and prevented fibrosis (70).

Regarding host defense, lung sensory neurons can regulate or activate the inflammatory response. CGRP prevents *Nippostrongylus brasiliensis* expulsion through the regulation of IL-33 and NMU-activated ILC2 (71). No reports have explored the role of fungal infection in the lungs with regards to neuroimmune crosstalk. Nevertheless, within the skin, *Candida albicans* can trigger sensory neurons *via* Dectin-1, which in response, release CGRP. This neuropeptide induces the release of IL-23 by CD301b+ dendritic cells, resulting in Th17 and γδ T cells mediated response, thus augmenting host defense (72, 73). During lung pneumonia induced by severe *S. aureus* infection, activated TRPV1+ neurons release CGRP in response to the bacterial infection, which in counterpart, regulate neutrophil and γδ T cell function. As a result, host defense is compromised, leading to bacterial proliferation and systemic dissemination resulting in lethal infection. CGRP blocking prevents neutrophil regulation, thus augmenting host defense (74). Similarly, in the context of skin infection with *S. pyogenes*, nociceptors suppress immune responses by releasing CGRP which inhibits neutrophil recruitment and subsequent killing of the bacteria. Botulinum neurotoxin A and CGRP blocking prevented suppression of neutrophil responses thus promoting host defense against *S. pyogenes* infection (75). On the other hand, Substance P and Gastrin-releasing peptide (GRP) release by sensory neurons can induce neutrophil chemotaxis and formation of neutrophil-neuron clusters (76). In the skin, optogenetic activation of sensory neurons in TRPV1-Ai32 mice elicited a type 17 immune response that induced significant neutrophil and T cell recruitment *via* release of CGRP. This response was protective in the context *C. albicans* and *S. aureus* infection and extended beyond the site of stimulation *via* a nerve-reflex arc (72). These pieces of evidence support the concept that neuromodulation of neutrophils depends on which neuropeptides are released as well as the inflammatory context. In response, neutrophils produce hypernociceptive factors, such as PGE2, ROS and sympathetic amines, increasing sensory neuron activation threshold (77).

In the context of virus infection, few studies have explored whether viruses can trigger nociceptors and the immunological consequences. During herpes simplex virus 1 infection, sensory neurons regulate neutrophil infiltration into the skin, concomitantly with the induction of a CD8 T cell-mediated antiviral response which limited the severity of tissue damage and restoration of skin homeostasis (78). Within the lungs, type I Interferon directly activates nociceptors, leading to neuronal depolarization (79). Recently, the world has experienced the consequences of the SARS-CoV-2 pandemic in which millions of people have succumbed to this infection. Furthermore, recent evidence has suggested that human sensory neurons express the receptors ACE2 and SCARF on their surface, which are used by SARS-CoV-2 for cell entry (80). Thus, it is plausible to suggest that sensory neurons can be infected and might explain some of the respiratory symptoms observed in COVID-19 patients, such as cough and loss of smell. Whether SARS-CoV-2 can be detected by sensory neurons through innate receptors and the physiological as well as the immunological consequences are still unexplored.

## COVID-19 and Nociceptors

**Figure 3:**
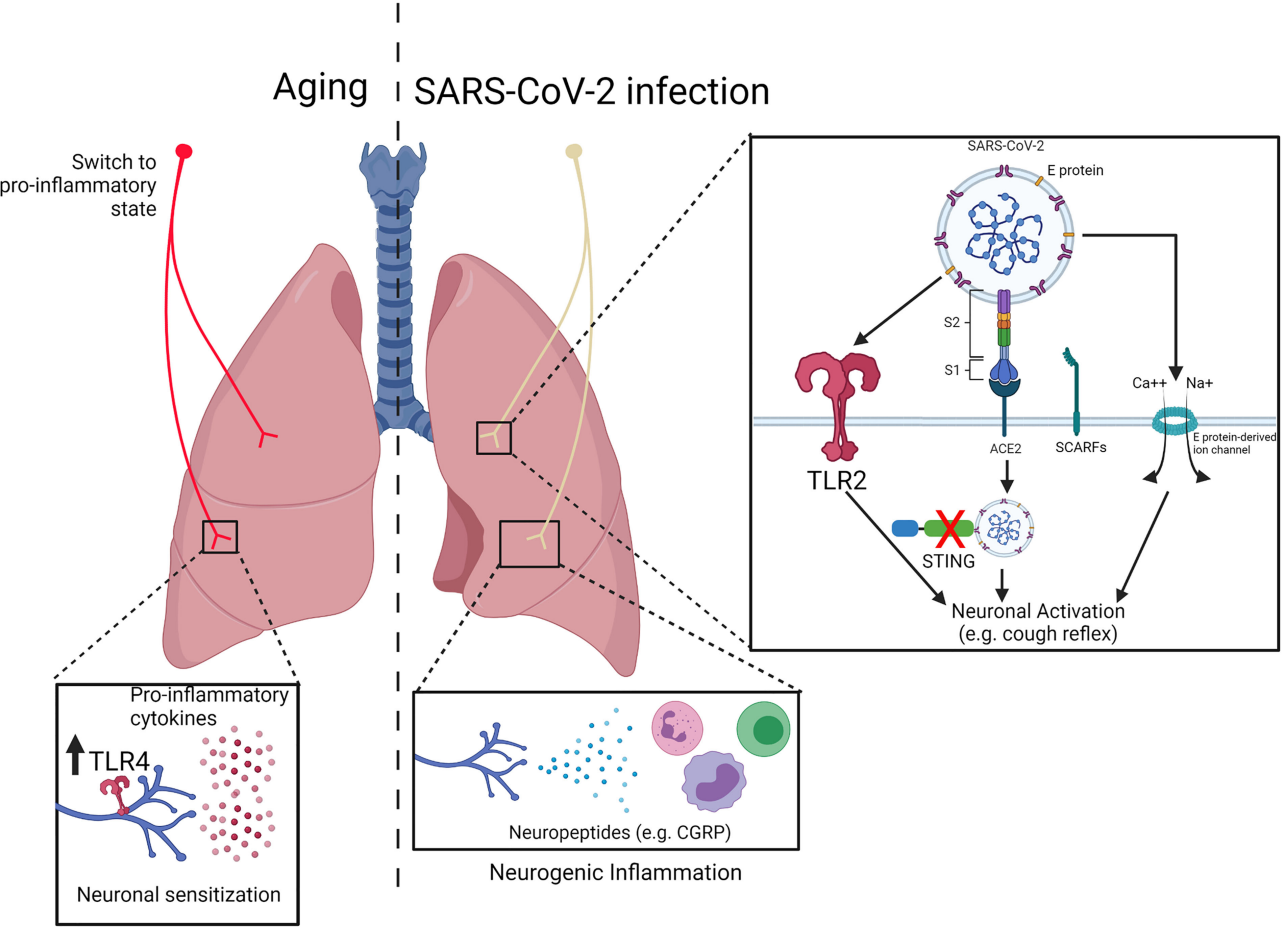
Impact of innate receptor expression in nociceptors during aging and COVID19. (Left) During aging, nociceptors switch from an anti-inflammatory to a pro-inflammatory state. Inflammaging results in an increased production of pro-inflammatory cytokines (e.g. TNF-α, IL-1β, IL-6) which might contribute to neuronal sensitization. Nevertheless, increase in the expression of innate receptors (e.g. TLR4) might contribute sensory neurons activation. (Right) SARS-CoV-2 has been described to trigger TLR2 as well as induce cell activation/death *via* E protein-mediated ion channel formation, which could lead to neuronal activation. Furthermore, expression of the receptors ACE2 and SCARFs by human dorsal root ganglia suggest that nociceptors can be a target of SARS-CoV-2 infection. Within the cell, the virus can block STING-mediated signaling, resulting in neuronal activation. Adapted from “Mechanism of SARS-CoV-2 Viral Entry”, by BioRender.com (2020). Retrieved from https://app.biorender.com/biorender-templates.

Since the beginning of the COVID-19 pandemic, SARS-CoV-2 infection has been described to cause airway damage, such as loss of multiciliated cells and alveolar disruption (81). Much of the damage in the lungs can also be due to overwhelming inflammation as well as reduced antiviral response, which leads to tissue injury and organ dysfunction. Single cell analyses reported impaired type I IFN response and increased expression of pro-inflammatory cytokines and chemokines in the lungs of COVID-19 patients (82–84). Furthermore, neuropathological features have been found in autopsies of COVID-19 patients in which an inflammatory infiltration as well as activated immune cells were found in specific regions of the central nervous system (85). Therefore, this suggests that neurological consequences might be mediated by neuroinflammation. Whether this neuroimmunopathological mechanisms occurs in the lungs and their consequences remains unanswered. The developments of models, such as hACE2 mice model (86) as well as organoids (87), has allowed a better understanding of the mechanisms through which SARS-CoV-2 infects and mediates COVID-19 pathology, including in the airways innervation. Furthermore, a ligand-receptor interactome analyzed bulk RNA-seq data from COVID-19 patients and human DRG gene expression. They found increased expression of pro-inflammatory chemokines (e.g. CCLs and CXCLs) within COVID-19 bronchoalveolar lavage fluid (BALF) as well as expression of their receptors by human DRGs, suggesting a connection between inflammation and sensory neurons activation (88). In the next paragraphs, we will cover some of the findings and discuss possible roles for sensory neurons in COVID-19. A representation of the relation between COVID-19 and nociceptors is represented by **Figure 3**.

To better understand the current pandemic, it is essential to evaluate the previous knowledge gained during the initial SARS pandemic. Earlier work with SARS-CoV demonstrated the neurotropic potential of SARS coronaviruses which correlated with neuronal and psychological abnormalities in SARS patients (89). SARS-CoV was shown to cause neuronal death in hACE2-transgenic mice and SARS-CoV patients had viral RNA in their cerebrospinal fluid (90–93). *In vitro*, human and rat neuron cell lines were also susceptible to infection by SARS-CoV (94). It was proposed SARS-CoV could gain entry into the central nervous system by infecting olfactory nerves and subsequently disseminating (91). Notably, viral E protein was shown to be important for neurotropism (95). E protein-deficient SARS-CoV, lacking E protein ion channel activity was less virulent and caused less inflammation in animal models (96, 97). The absence of E protein can affect viral particle release from infected cells and result in decreased viral loads and inflammation. However, the ion channel activity of the E protein could also potentially directly affect the neuronal membrane, resulting in neuron death and neurogenic inflammation, which would not be present during infection with E protein-deficient SARS-CoV. Hence, the combination of direct neuron infection and the effects of viral proteins of neuronal cells, provide some hints into the neurological symptoms and potentially places neurons as drivers of inflammation during infection with coronaviruses.

A common feature manifested by COVID-19 patients is the loss of taste and smell (98). Similar to SARS-CoV, it suggests that olfactory sensory neurons can be affected by SARS-CoV-2 infection, resulting in loss of function and/or neuronal death. Indeed, in olfactory mucosa brush cytological sampling from COVID-19 patients with loss of smell, SARS-CoV-2 was detected in sensory neurons as well as non-neuronal sensory epithelial cells. Staining of cleaved caspase-3 in these cells suggests cell death induction (99). Curiously, single-cell analyses have shown that olfactory sensory neurons do not express ACE2 and TMPRSS2 (100); nevertheless, Neuropilin-1, a receptor for furin-cleaved substrate, can facilitate SARS-CoV-2 infection within olfactory epithelium (101). Furthermore,SARS-CoV-2-derived E protein can form a pH sensitive ion channel at the membrane of several cell lines, which causes their death and an overwhelming secretion of cytokines and chemokines (102). As mentioned before, sensory neuron activation through TRP channels induces ion influx and neuron firing, resulting in release of neuropeptides. There is no evidence of direct olfactory sensory neuron activation by SARS-CoV-2; nevertheless, it is plausible that SARS-CoV-2-derived E protein could form a pore in the membrane of sensory neurons, resulting in cellular death and exaggerated neuropeptides release, which would explain the symptoms mentioned above.

Some COVID-19 patients manifest persistent symptoms even months after the initial insults. These symptoms, known as post-acute COVID-19 syndrome, include recurrent cough which indicates that sensory neurons can be chronically activated (103, 104). Although this reflex contributes to viral spread during acute infection through the dispersion of virus-containing droplets from the airways, it is less clear how and why chronic cough develops once the virus is eliminated. Blocking cough in the acute setting is a potential mechanism to prevent viral spread and this may result in alleviating chronic symptoms (105). Recently, the innate receptor TLR2 was found to recognize SARS-CoV-2 E protein, resulting in an inflammatory response (106). Lung nociceptors express TLR2 (18), which suggests that they could also be activated by SARS-CoV-2 *via* this innate receptor and trigger neuronal activation. Additionally, COVID-19 patients can have an overwhelming production of several cytokines. A large study correlated serum levels of TNF-α and IL-6 with disease severity and death of COVID-19 patients (107). Despite the absence of evidence, it is possible that these cytokines could directly activate lung sensory neurons and trigger cough reflex as well as contribute to neurogenic inflammation. Even though production of antibodies is one of the most effective immuno-protective mechanisms of our body, recent evidence has demonstrated a deleterious role, such as the generation of autoantibodies against type I IFN, which prevents viral clearance (108). Furthermore, in the context of airway hypersensitivity, increased levels of IgE are correlated with overwhelming pulmonary mast cell degranulation and histamines release, resulting in bronchoconstriction and blockage of the respiratory tract (109). Lung nociceptors express functional FcϵR1, which triggers neuronal activation (57). Thus, production of IgE during SARS-CoV-2 infection could be contributing for sensory neurons activation along the respiratory tract and initiate cough. Omalizumab, an IgE blocker currently approved for the treatment of asthma, nasal polyps and urticaria, has been suggested as a promising COVID-19 treatment to prevent excessive airway hypersensitivity response. If IgE is proven to induce sensory neuron-mediated neurogenic inflammation, this treatment could be a promising candidate for COVID-19 treatment (110).

Human dorsal root ganglia express ACE2 and SCARFs receptors, which are used by SARS-CoV-2 for cell entry (80). Within the sensory neuron, SARS-CoV-2 might interact with intracellular innate receptors, leading to activation or regulation. Future studies will clarify if this recognition occurs. Nevertheless, as mentioned before, nociceptors express STING, which its activation results in regulation of nociceptive sensation *via* IFN-signaling (39). Importantly, SARS-CoV-2-derived ORF3a has been found to block STING activation (111). It is possible that blockage of STING signaling by SARS-CoV-2 would not only impair anti-viral immune response, but would also increase nociceptor sensitization, thus leading to cough reflex and viral spread. Indeed, pharmacological activation of STING using small-molecule agonists prevents SARS-CoV-2 infection *via* IFN-signaling (112). If proven that SARS-CoV-2 induces nociceptor sensitization *via* STING blockage, the treatment with agonists could potentially prevent nociceptor activation and the cough response.

Post-acute COVID-19 syndrome affects both the respiratory and nervous systems (104). Increased levels of neuro-axonal damage marker in the peripheral blood of COVID-19 patients suggests directly or indirectly injury to neurons (113, 114). Whether lung sensory neurons could be chronically damaged by SARS-CoV-2 infection is poorly described. Therapeutic approaches targeting TRPV1 sensory neurons using resiniferatoxin, a potent TRPV1 agonist, have been suggested for use in patients with severe COVID-19 (115). SARS-CoV-2 has neurotropic potential suggesting nociceptors could be a reservoir for the virus and drive symptoms. Hence, ablation of TRPV1+ nociceptors could be beneficial as a therapeutic strategy. Depletion of nociceptors that could be chronically activated and potentially drive inflammation, could modulate the immune response, and help improve outcomes. Moreover, blocking CGRP for example, by means of anti-CGRP antibodies used for migraine treatment, could prevent the excess neuropeptide release that might contribute to the hyperinflammatory response in patients with severe COVID-19 (116). TRPV1 neuron depletion and CGRP antagonism has been proven to be favorable in animal models of bacterial pneumonia as TRPV1+ sensory neurons suppressed protective immune responses through CGRP release (74). However, the effectiveness of these strategies in the context of respiratory viral infections remains undefined. It is also possible that in the setting of COVID-19 hyperinflammation, the absence of immune modulation through neuropeptide release by sensory neurons could have a detrimental effect. Levels of CGRP were found to be decreased in COVID-19 patients. Despite the absence of correlation with disease severity, these findings suggest that neuroinflammation might influence COVID-19 outcome (117). Yet, TRPV1+ nociceptors could also become more pro-inflammatory with age, in which case it would make sense to block afferent signaling from TRPV1 sensory neurons in elderly COVID-19 patients (118).

## Aging, Nociception, and Implications For Immune Function

Similar to many diseases, lung infections including bacterial pneumonia, and COVID-19, older patients have increased susceptibility and worse clinical outcomes. Although data is emerging, it remains unclear if and how an aged neuronal sensory system contributes to clinical differences in diseases that target the lung and breathing. Aging is a natural and continuous part of everyday life. Even barring pathological processes such as degenerative diseases, cancers, and/or lifestyle-related illnesses (e.g. high blood pressure, diabetes etc.), aging brings about significant changes to physiological functions. Such changes have been well-characterized elsewhere, from age-related fertility decline to decreased renal and hepatic function, no organ system is left untouched (119–121). Given our current knowledge of associations between the immune and nervous system, innate sensing by nociceptors likely plays a major part in facilitating bidirectional signaling involved in pain sensation and inflammation. What is less clear is the mechanism by which neuroimmune interactions influence pulmonary host defense as we age. A representation of the possible role of aging in nociceptors is represented by **Figure 3**.

Briefly, it has been known for more than three decades that aging brings about widespread changes to the nervous system (122). Neurocognitive declines seen in dementia/Alzheimer’s and a host of other maladies are increasingly becoming linked with age-related demyelination, neuronal density and synapse structure changes (123, 124), as well as alterations in neuropeptide release (125). These also coincide with changes to pain perception and nociceptive pathways (126, 127). Similarly, with regards to lung function, development ends at around 20-25 years of age (128). Starting in the third and fourth decade of life, respiratory function gradually begins to decline because of anatomical changes in the lung parenchyma and weakening respiratory muscles (129). Such changes can be readily measured and tracked with lung function tests and typically manifest in symptoms such as shortness of breath (especially with exertion), chest pain, and chronic cough (115). Additionally, older individuals tend to be at a higher risk of developing pneumonias and other respiratory infections (130).

A key factor for this susceptibility is age-related immune dysregulation. More specifically, a systemic increase in baseline inflammation stemming from innate immune system activation despite the absence of any immunological threats (130). Indeed, the immune system is equally vulnerable to deteriorations with age – a phenomenon termed as ‘inflammaging’. Inflammaging’s prevailing phenotype is one of elevated proinflammatory cytokines (particularly IL-1β, IL-6, and TNF-α) and aberrant leukocyte trafficking (131). For instance, in murine models, neutrophils from aged mice adhere to and breach the vascular endothelium at a higher frequency than those of young mice. Re-entering systemic circulation, these neutrophils have the potential for eliciting peripheral organ injury as evidenced by their contribution to enhanced vascular leakage within the lung (132). Peculiarly, despite this heightened immunological activation, inflammaging is closely associated with a dampened ability to respond to pathogenic threats or tissue injury (termed immunosenescence) (133).

The impact of aging in innate receptors expression is controversial. Expression of TLR1, 2, 3, 4, 5, and 11 and their downstream signaling pathway molecules MyD88 and Phospho-IRF-3 were reported to be significantly elevated in aged renal tissues sourced from rats (134). Meanwhile, other studies have reported opposite observations in which expression of TLR1 and TLR4 decreased in human monocytes with aging (135). Similar trends have been observed in other immune cell types such as macrophages and plasmacytoid dendritic cells which show significantly decreased TLR1-TLR9 expression in aged mice and reduced TLR7 or TLR9 in older humans, respectively (136). These discordant findings have made it difficult to ascertain the precise effect(s) of aging on innate receptors over the lifespan.

While it is not entirely clear which mechanisms influences the immune system as we age, recent findings pertaining to TRPV1 nociceptor function (particularly in the context of aging) have provided intriguing clues. In addition to their canonical role as sensors of noxious stimuli, TRPV1 neurons have been shown to possess important immunoregulatory roles. As mentioned before, lung nociceptors suppress neutrophil recruitment and surveillance during infection *via* the release of neuropeptides, such as CGRP (74). Similar findings have been reported in the context of systemic inflammatory response syndrome (SIRS); whereby TRPV1 nociceptors plays an immunosuppressive role by inhibiting production of TNF-α (118). Thus, the anti-inflammatory phenotype of TRPV1 nociceptors appears to be conserved across various illnesses.

Interestingly, the anti-inflammatory actions of TRPV1 may reverse with aging. Wanner et al. found that pharmacological or genetic ablation of TRPV1 in young mice resulted in increased mortality during aseptic SIRS. Whereas the opposite occurred for aged mice – with TRPV1 antagonism in aged mice resulting in greater survival during aseptic sepsis (118). They also noted decreased serum TNF-α in TRPV1 knockout mice when compared with age-matched wildtype littermates; suggesting that TRPV1 inhibition of TNF-α production may also be reversed with aging. One possible explanation for why this may occur is that neurons that previously possessed anti-inflammatory properties begin to transition into a more proinflammatory state through aging. Thus, when their effects are blocked in older mice experiencing sepsis, excessive neurogenic inflammation is decreased, preventing death (118). Indeed, levels of neuropeptides, such as CGRP and VIP, are dysregulated in aged mice, which impacts physiological functions, like metabolic homeostasis (125, 137). Nevertheless, the mechanisms behind aging-induced nociceptors modulation and the influence in the immune system are poorly described.

Chronic cough is more prevalent in the elderly population, potentially implicating lung sensory neurons sensitization as we age (138). This could be explained by the increased levels of pro-inflammatory cytokines which could chronically activate lung nociceptors and trigger cough reflex. To the best of our knowledge, there is no evidence showing whether sensory neurons can upregulate innate receptors expression as we age neither the impact on the immune system. Nevertheless, upregulation of TLR4 expression in hippocampal neuron of aged mice leads to higher response upon LPS injection, resulting in neuronal death (139). It suggests that neurons can upregulate innate receptors as we age. Future studies will elucidate whether aging increases expression of innate receptors in nociceptors, potentially contributing to neurogenic inflammation in elderly individuals.

It is well-established that individuals with underlying medical conditions are at a higher risk of complications and/or death from COVID-19. Many of these underlying conditions such as COPD and chronic kidney disease are becoming increasingly linked with defective neuroimmune interactions (140, 141). However, what is less certain are the underlying age-related mechanisms driving differences in COVID-19 disease severity. Numerous hypotheses for the link between COVID-19 lung injury and aging have been posited. For example, greater pre-existing immunity to commonly circulating human coronaviruses in adults and geriatric patients may facilitate COVID-19 cell entry and viral replication *via* non-neutralizing antibodies (142). Similarly, age-related increases in expression and affinity of ACE2 receptors may be another driving factor in severe COVID-19 (143). A potential neuroimmune explanation may be that if neurons are indeed a target for the virus, it is then plausible that in the elderly, the neurogenic inflammation that results from COVID-19 infection combined with immunosenescence and inflammaging guides the severe natural history of disease. Furthermore, higher levels of neuron damage markers are found in the blood of elderly COVID-19 patients, suggesting that neuronal infection and damage might contribute to increased susceptibility (113). Therefore, we suggest that therapeutically blocking neuroimmune mediated inflammation could lead to similarly reduced mortality and better clinical outcomes in COVID-19 patients, especially in elderly individuals.

## Concluding Remarks

The neuroimmune crosstalk in the lungs is still poorly described. Notably, the mechanisms through which lung sensory neurons are activated during pulmonary diseases and the relevance of innate receptors remain even less well defined. Here, we review recent evidence which highlights the importance of innate receptors in lung nociceptors with regards to their expression as well as the physiological and immunological consequences of their activation *via* these receptors. Furthermore, we also describe the possible involvement of nociceptors during SARS-CoV-2 infection as well as their functional changes and activation during aging. Whether the increased susceptibility of elderly patients to SARS-CoV-2 infection is mediated by lung nociceptors remains an unanswered question. Nevertheless, recent evidence is contributing to our understanding of the mechanisms behind pulmonary neuroimmune crosstalk, which will open the possibility for promising therapeutic targets for lung diseases.

## Author Contributions

All the authors contributed with intellectual discussion, figures preparation, and manuscript writing.

## Funding

This work was supported with an operating grant from the Canadian Institutes of Health Research (RS-342013), a Beverley Phillips Graduate Scholarship (to CH) and a tier II Canada Research Chair in Pulmonary Immunology, Inflammation and Host Defense (to BY).

## Conflict of Interest

The authors declare that the research was conducted in the absence of any commercial or financial relationships that could be construed as a potential conflict of interest.

## Publisher’s Note

All claims expressed in this article are solely those of the authors and do not necessarily represent those of their affiliated organizations, or those of the publisher, the editors and the reviewers. Any product that may be evaluated in this article, or claim that may be made by its manufacturer, is not guaranteed or endorsed by the publisher.
